# Global Outreach of a Locally-Developed Mobile Phone App for Undergraduate Psychiatry Education

**DOI:** 10.2196/mededu.4179

**Published:** 2015-06-08

**Authors:** Melvyn WB Zhang, Christopher CS Cheok, Roger CM Ho

**Affiliations:** ^1^ National Healthcare Group Singapore Singapore; ^2^ National Addiction Management Service Institute of Mental Health Singapore Singapore; ^3^ National University of Singapore Singapore Singapore

**Keywords:** psychiatry, education, eLearning, mobile phone apps, mobile phones, feasibility, proof of concept

## Abstract

**Background:**

Over the past decade, there have been massive developments in both Web-based and mobile phone technologies. Mobile phones are well accepted by students, trainees, and doctors. A review of the current literature has identified the following specialties that have used mobile phones in education: pediatrics, ophthalmology, nephrology, plastic surgery, orthopedics, pharmacology, and urology. However, to date, there are no published papers examining the application of the latest mobile phone technologies for psychiatry education internationally.

**Objectives:**

The main objectives of this study are (1) to determine the feasibility and receptiveness of a locally-developed psychiatry mobile phone app and user perspectives (both quantitative and qualitative) towards it, and (2) to determine the receptiveness of a locally-developed app for psychiatry education internationally.

**Methods:**

A Web-based app that contained textbook contents, videos, and quizzes was developed using HTML5 technologies in 2012. Native apps were subsequently developed in 2013. Information about the apps was disseminated locally to Singaporean medical students, but the respective native apps were made available on the app stores. A user perspective survey was conducted locally to determine student’s perception of the app.

**Results:**

From the inception of the app until the time of preparation of this manuscript, there have been a cumulative total of 28,500 unique visits of the responsive HTML5 Web-based mobile phone app. There have been a cumulative total of 2200 downloads of the Mastering Psychiatry app from the Apple app store and 7000 downloads of the same app from the Android app store. The initial user perspective survey conducted locally highlighted that approximately a total of 95.2% (177/186) of students felt that having a psychiatry mobile phone app was deemed to be useful. Further chi-squared analysis demonstrated that there was a significant difference between males and females in their perception of having textbook contents in the mobile phone app (χ^2^_4_=12.9, *P*=.0012).

**Conclusions:**

To the best of our knowledge, this is the first study to demonstrate the feasibility and global acceptance of a local, self-designed educational app for psychiatry education. Whilst the current research has managed to demonstrate the feasibility and acceptance of such an app, future studies would be warranted to look, in-depth, into whether there are cultural differences in terms of perceptions towards having such an app in psychiatry and what contents different cultures and cohorts of students might want within an app.

## Introduction

Over the past decade, there have been massive developments in both Web-based and mobile phone technologies. It was perhaps the release of Apple’s iPhone in 2007 and the launch of the Apple App store in July, 2008 that was pivotal in causing a huge change in the way the world uses mobile phone devices. Mobile phones with app capabilities are considered to be a new generation of mobile technology, that are equipped with immense computing capabilities allowing individuals to have constant access to the internet and make use of various apps [1].

Previous studies have looked into medical students and trainee’s ownership and perspectives towards mobile phone usage. In 2012, a questionnaire-based survey was distributed amongst the interns in the Republic of Ireland [2]. The survey demonstrated that mobile phones were widely adopted and accepted and that they were being used by interns to aid them in performing their daily duties. The survey highlighted that the most popular app that was commonly used was that of the British National Formulary app [2]. Other studies have highlighted similar results, in that there was a high usage rate of mobile phones and the associated apps amongst medical students and junior doctors, and other studies have also found that iPhone users tend to own more apps [3]. Previous studies indicate that students and junior doctors make use of the app for an estimated 30 minutes each day [3]. Given the results of the previous questionnaire surveys, it is apparent that mobile phone technologies are well accepted by students, trainees, and doctors. It would be of interest to determine how several specialties have made use of a hybrid of online and mobile device technology in educational settings.

A review of the current published literature on several databases has identified the following specialties to be using mobile phone technologies in education: pediatrics, ophthalmology, nephrology, plastic surgery, orthopedics, pharmacology, and urology. For example, in pediatrics, a mobile phone neonatal intubation app was deployed to enhance overall intubation skills [4]. In ophthalmology, a cumulative total of 342 apps have been identified to be of value in terms of enhancing clinical skills [5]. In nephrology, several online Web-based resources were identified to be of value for medical students and residents to augment their knowledge with regards to the complications of chronic kidney disorders [6]. In plastic surgery, 16 apps that are of educational value to the plastic surgeon have been identified [7]. It is thus of interest to us to determine to what extent psychiatry, as a discipline, has embraced online, Web-based, and mobile phone technologies for educational needs of psychiatry medical students and residents. A literature search revealed that the most recent apps of Web-based technology was that of the usage of virtual worlds for role-play simulation in child and adolescent psychiatry [8], the usage of telemedicine for peer-to-peer psychiatry learning between medical students in the United Kingdom and Somaliland [9], the usage of stimulation for performance evaluation in psychiatry [10], and the usage of virtual patients as training tools to teach clinical interviewing skills [11,12]. A search through the existing published literature using the keywords “psychiatry, smartphone, education” did not yield any published papers to date that examined the app of the latest Web-based and mobile phone technologies for psychiatry education.

We hope to make use of the latest Web-based and mobile phone technologies in implementing both a Web- as well as a native mobile phone-based psychiatry textbook companion for undergraduate students in psychiatry, as a means of overcoming the limitations in the current literature. In addition, we hope to be able to determine local and international users' receptiveness towards such an innovative methodology of acquiring psychiatry knowledge.

The main objectives of this study are (1) to determine the feasibility and receptiveness of a locally-developed psychiatry mobile phone app and user perspectives (both quantitative and qualitative) towards it, and (2) to determine the receptiveness of a locally-developed app for psychiatry education internationally.

## Methods

A newly written textbook (jointly written by the authors MWBZ and RCMH of this study) that integrated both local (Singapore) and United Kingdom clinical guideline was initially crafted in 2011. The core textbook contents are comprised of chapters, which include subjects in the areas of psychopathology, clinical interview, formulation and management, schizophrenia and psychotic disorders, mood disorders, anxiety disorders, personality disorders, substance misuse and dependence disorders, eating disorders, psychiatric emergencies, psychotherapies, sleep disorders, psychosexual disorders, somatoform and dissociative disorders, consultation liaison psychiatry, old age psychiatry, child and adolescent psychiatry, and forensic psychiatry and psychiatry ethics. As the book was self-published by the authors of this study, the copyrights of the chapters belonged to the authors and hence no permissions were required to reproduce the materials in the mobile phone app. In addition, the authors filmed videos demonstrating assessment methodologies for the various psychiatric disorders locally in Singapore (Textbox 1).

**Filmed videos demonstrating assessment methodologies for various psychiatric disorders**

Videos

1. Psychosis: history taking
2. Depression: history taking
3. Anxiety: history taking
4. Explanation of antidepressants
5. Explanation of cognitive behavioral therapy
6. Assessment of borderline personality disorder
7. Suicide risk assessment
8. Explanation of electro-convulsive therapy treatment
9. Frontal lobe examination
10. Mini mental state examination
11. Explanation of lithium therapy
12. Explanation of neuroleptic malignant syndrome
13. Violence risk assessment
14. Explanation of dementia medications
15. Sleep disorder assessment

The core textbook materials, as well as the videos, were then integrated into a Web-based mobile phone app. The Web-based mobile phone app was programmed using an online app builder and a blogging website using both HTML5 and Java-script coding. Videos were stored online on a video-hosting website (Vimeo) [13], and embedded within the Web-based mobile phone app. In addition, a questionnaire-based quiz that contains both multiple choice questions and short answered questions was crafted using an online questionnaire builder and integrated into the Web-based app. Prior to the launch, the usability of the app was evaluated across several different computing platforms to ensure that the system was robust.

In 2013, the authors MWBZ and RCMH were offered an educational grant for the development of a native Apple- and Android-based mobile phone app. The English language version of the Apple IOS and Android Play apps were made available for free on the app stores in late 2013. Given that the apps were made available for free downloads, no proceeds arose from their downloads.

Information about the app was disseminated via printed materials locally in Singapore, as well as by means of a short demonstration to undergraduate students at the National University of Singapore, Yong Loo Lin School of Medicine on the first day of their clinical psychiatry posting. With ethics approval from the National University of Singapore, a user perspective survey was administered to students immediately after the end of their posting test to determine user’s perspectives towards the usefulness of such an app.

## Results

### Download Statistics

**Figure 1:**
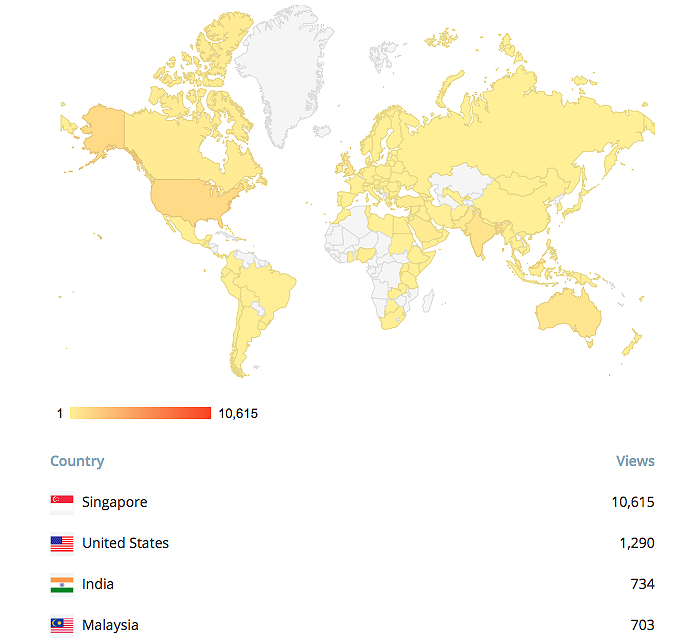
Geographical map of the utilization of the web-based app since its inception.

The Web-based mobile phone app, Mastering Psychiatry [14], was launched on July 15, 2012 and from the inception of the app until the time of preparation of the manuscript, there have been a cumulative total of 28,500 unique visits of the responsive HTML5 Web-based app. The majority of the users were from Singapore (68.01%, 19,383/28,500), followed by the United States (5.22%, 1487/28,500) and Malaysia (3.31%, 942/28,500). The geographical utilization of the portal since inception until the time of preparation of this manuscript is shown in Figure 1.

**Figure 2:**
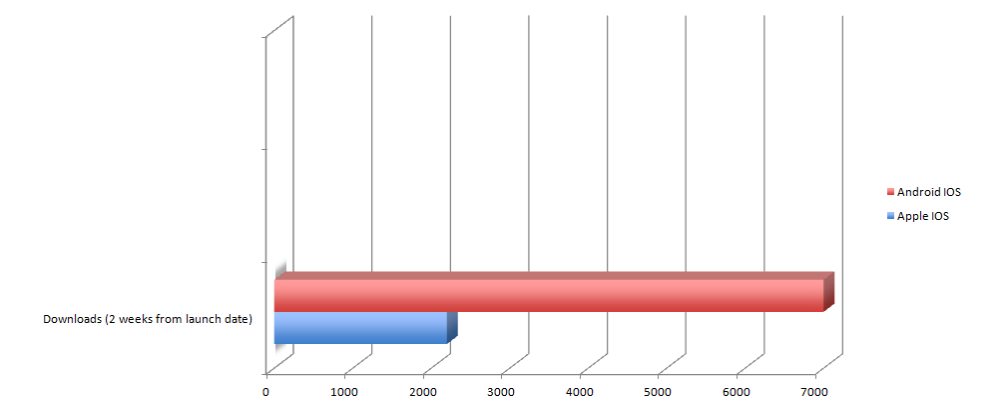
Cumulative total number of downloads from each of the respective app stores.

With regards to the utilization of the native apps, there have been a cumulative total of 2200 and 7000 downloads of the Mastering Psychiatry app from the Apple and Android app store, respectively (Figure 2). To our knowledge, the native app has been granted a score of 4+ out of 5 on the Apple app store, whilst on the Android app store, it has been granted a score of 4.5 out of 5. A total of 161 users have rated our app on the Android store and a cumulative total of 88.8% (143/161) of the users have given the app a score of ≥3. Some of the qualitative feedback made available on the Android store was that the app was deemed to be a great book for beginners and that it was an excellent app. Some users communicated technical issues pertaining to the usage of the app on some devices to the authors and the app developers.

### User Perception Survey and Focus Group Analysis

A cumulative total of 185 students voluntarily participated in the user perspective survey. The mean age for males was 22.3 years (SD 0.8) and that for females was 22.0 years (SD 0.4). The majority of the students (53.3%, 121/227) used an Apple IOS device, whereas 21.6% (49/227) of the students used an Android device. The majority of the students (66.7%, 124/168) had between 1-5 medical apps in their mobile phones. The medical apps they had previously downloaded were mainly used for educational purposes as well as for use in the clinics and wards. Table 1 shows the baseline demographic information and the statistical analysis that have been conducted to evaluate the differences between the genders.

In terms of student’s perception about the utility of the app, a total of 95.2% (177/186) of the students indicated that having a psychiatry mobile phone app would be useful. The majority of the students wanted the app to contain textbook content and clinical videos and found these features particularly useful. Students perceived that an event management system within the app would be helpful for coordinating the tutorials. A total of 57.1% (105/184) of the students agreed that the app for psychiatry was helpful, and 71.4% (132/185) of the students agreed that a mobile phone app would be a good companion to a traditional textbook.

Further chi-squared analysis demonstrated that there was a significant difference between males and females in their perception of having textbook contents in the app (χ^2^_4_=12.857, *P*=.0012). There were no demonstrated significant differences between the genders in terms of their perception of having an app in mastering psychiatry, having clinical videos in the apps, having revision lecture videos in the apps, having text messaging notification services in the app, and in terms of the usefulness of the app, and whether the app was a good companion to a book. Table 2 shows the statistical analysis of the gender comparisons with respect to their perceptions to the individual app features.

A focus group was conducted with a cumulative total of 5 students (n=5). Thematic analysis was conducted and qualitative feedbacks are summarized in Multimedia Appendix 1.

**Table 1:** Baseline demographic information and statistical analysis conducted to evaluate the differences between the genders (N=185).

Demographic variables		Male	Female	Statistical data	*P* value
Age, years (SD)		22.3 (0.8)	22.0 (0.4)	^a^ *t*_176_ =2.7	.008
Average monthly income, dollars (SD)		4214.36 (2778.33)	4583.33 (2800.30)	*t*_18_ =-0.3	.789
**Mobile phone ownership, %**				^b^χ^2^_5_=6.0	.307
	None	1.3%	0.0%		
	iPhone	27.9%	25.2%		
	Google Android	10.6%	11.1%		
	iPad	6.2%	4.4%		
	Android Tablet	2.7%	0.9%		
	Laptop/notebook computer	5.3%	4.4%		
**Medical-related apps, %**				χ^2^_4_=3.3	.508
	None	13.5%	8.1%		
	1-5 apps	33.5%	33.0%		
	6-10 apps	4.9%	3.2%		
	11-15 apps	0.5%	1.1%		
	≥15 Apps	1.6%	0.5%		
**Purpose of medical-related app, %**				χ^2^_4_=5.7	.220
	Education- revision	9.7%	5.2%		
	Education- learning	12.3%	14.9%		
	Clinical (wards)	14.9%	16.0%		
	Clinical (clinics)	10.1%	10.4%		
	Others	4.1%	2.2%		
**Frequency of medical app usage, %**				χ^2^_3_=2.1	.560
	Rarely	30.7%	25.6%		
	2-3 times per week	13.1%	12.5%		
	1-2 times per day	5.7%	6.3%		
	≥3 times a day	4.5%	1.7%		
**Time spent on medical app per day, %**				χ^2^_6_= 5.0	0.549
	None	19.0%	15.6%		
	1-10 mins	22.9%	17.9%		
	11-20 mins	6.1%	5.6%		
	21-30 mins	3.9%	3.9%		
	31-60 mins	2.2%	1.1%		
	1-24 h	0.0%	1.1%		
	≥24 h	0.6%	0.0%		

^a^ *t* test

^b^Chi-square test

**Table 2:** Comparison between the genders in terms of their perceptions to the individual app features.

Perspectives	Males, %					Females, %					Chi-square^a^
	Absolutely useless	Useless	Of Some use	Useful	Very Useful	Absolutely useless	Useless	Of Some use	Useful	Very Useful	
Mobile phone app to learn psychiatry	1.1	2.7	28.1	16.8	5.4	0.0	1.1	22.2	15.1	7.6	5.0
Textbook content in app	2.2	3.8	17.4	22.3	8.2	0.0	0.5	11.4	22.8	11.4	12.9
Clinical OSCE videos in app	0.5	4.3	13.4	24.2	11.8	0.5	2.2	12.9	18.8	11.3	1.3
Revision lecture videos in app	1.6	4.8	15.1	22.0	10.8	0.5	2.7	11.3	18.8	12.4	2.5
SMS notification/event management services in app	1.6	2.7	17.3	18.4	14.6	0.5	2.2	9.7	20.0	13.0	3.9
Usefulness of app for psychiatry	4.9	23.9	22.8	2.2	0.5	5.4	22.8	16.8	0.5	0.0	3.7
Good companion to book	9.8	29.3	9.2	4.3	1.6	10.9	21.2	11.4	1.6	0.5	5.0
Recommended app for other medical fields	14.7	32.1	6.0	0.5	1.1	10.9	26.6	7.6	0.5	0.0	3.7

^a^Chi-square values reported as χ^2^_4_

## Discussion

### Principal Findings

To the best of our knowledge, this study is one of the first to demonstrate the success of the implementation of mobile phone technologies for educational needs in psychiatry. The initial findings demonstrate the feasibility and acceptance of mobile phone apps for psychiatry in Singapore, as well as the feasibility and acceptance of psychiatry-focused apps globally. Our initial findings show that Asian students are amenable to using an online portal for their educational needs in psychiatry. In addition, a significant group of Asian students are amenable to trying newer modalities of technology, such as mobile phone technologies, to help them fulfill their mobile educational needs. The user perspective survey results show that a high proportion of students would like an educational psychiatry app to contain textbook-based content, clinical OSCE videos, and an event notification service. A high proportion of students concurred with the perception that a mobile phone app would be helpful in psychiatric education and that a mobile device could be a viable alternative to a traditional textbook. We postulate that the gender differences identified with regards to having textbook content in apps might be due to differences in learning methods between the genders. Of importance, no significance differences were found between the genders in the other domains, highlighting that the other materials included appealed to both genders and did help them with mastering a specialized topic matter.

The usefulness of mobile phone apps for education has been supported by prior research. Tripathi et al [15] conducted a review of relevant apps for neurosurgery on the respective app stores and highlighted that students and medical doctors preferred apps that have links to scoring systems, operative illustrations, as well as textbook-based contents. In consideration of the previous findings, we postulate that the current success of our app internationally might be due to the fact that the app offers students not only access to textbook-based materials, but also access to other materials such as videos and questionnaire quizzes. These might be relevant and deemed useful with regards to helping students to acquire the necessary knowledge in psychiatry.

Another recent study published in 2014 by Heeyoung Han et al [16] examined medical students’ online learning technology needs. In that study, the authors developed a 120-item survey in collaboration with the New Technology in Medical Education Committee at Southern Illinois University to investigate students’ perceptions of their online learning technology needs. The results of their study concurred with our findings with regard to students’ perceptions of their online learning technology needs. In their study and the current study, both samples perceived multimedia tools, scheduling tools, and communication tools to be useful educational technologies for their learning. Similar to our findings, their study showed that students in their clinical clerkship years perceived mobile devices to be useful for their learning.

Perhaps the closest correlation with our current study is by Waldmann and Weckbecker [17]. They designed a Web-based app to help teach their medical students about primary care guidelines and found that amongst their group of 14 student testers, the majority made use of the app more frequently, and also made use of their free time to study the guidelines. They concluded that their self-designed mobile phone app has helped to create interest amongst student and has helped them to acquire valuable knowledge. Similarly, our self-created Web-based and native app has enabled students to learn on the go, as well as help to augment their learning needs in psychiatry.

The main strength of the current study is the demonstration of the feasibility of implementation of a mobile phone app for psychiatry both locally and internationally. The current study has also managed to demonstrate that local Singaporean students perceive mobile phone apps in psychiatry positively. This study also consistent with some of the findings of other studies with regards to the usage of apps for education.

### Limitations

There are several limitations to the current study. We acknowledge that while there is a good number of viewership of our Web-based app, we do not have the absolute number of individual users who have downloaded the app, as we are limited by the database being able to only track individual unique access. This information, in conjunction with the platform that users view the app, is crucial in terms of designing future educational apps, as well as planning future studies. In addition, our perspective survey has only been administered to a local cohort of students and might not be entirely representative of the views of the global audience. To mitigate this limitation, we would need to find liked-minded individuals from organizations overseas to collaborate and perform a comparative study with regards to user perception of our educational app.

### Conclusions

This study is one of the first to demonstrate the feasibility and the global acceptance of a local, self-designed educational app for psychiatry education. Whilst the current research has managed to demonstrate the feasibility and acceptance of such an app, future studies are warranted to look in-depth into whether there are cultural differences in terms of perceptions towards having such an app in psychiatry and what contents different cultures and cohorts of students might want within an app.

## Appendix

Multimedia Appendix 1 Thematic analysis of how the app has helped learning in psychiatry.
