# Supplementary material for: Global Outreach of a Locally-Developed Mobile Phone App for Undergraduate Psychiatry Education
Source: JMIR Med Educ. 2015 Jun 8;1(1):e3. doi: 10.2196/mededu.4179 (PMC5041350; doi:10.2196/mededu.4179)
Supplement: Multimedia Appendix 1 [file mededu_v1i1e3_app1.pdf]

Themes	Qualitative feedback
Improvement in learning outcomes and facilitating acquiring needed knowledge in psychiatry	“The Mastering Psychiatry application was useful as the content was comprehensive and covered the core “must know and must see” conditions in Psychiatry thoroughly while providing an adequate introduction to lesser commonly encountered but nonetheless relevant conditions. A shortcoming of some of the textbooks was that core conditions were sufficiently elaborated but lacked information on other miscellaneous conditions seen in clinical practice. The one-stop online accessibility enabled one to read up without having to cross-reference several other textbooks, which is a cumbersome process”<br><br>“The website has an update column which I find useful in helping users to keep up to date with the changes made in the textbook and the application”<br><br>“I found the availability of online MCQ questions very useful. It provided me with a way to practice these questions using pockets of time while on the move. Other than convenience, these questions also helped me to consolidate my learning and identify any gaps that I may have.”<br><br>“Putting the contents in the form of e-book also enabled us to search for the contents we want easily, thus making it more efficient for our learning, and we are able to identify the more important areas to focus on. This platform is usable with such ease that I would be able to revise my psychiatry contents without any issues when I feel that I am deficient in skills or knowledge in the field of psychiatry.”
Enriched learning	“Furthermore, the multimedia dimension of the application /website helped me to learn signs and soft skills virtually without being by the bedside. For example, some of the signs in Psychiatry are difficult to describe in words but become apparent and easily understandable once observed (either during a live interview or video content, which the application provides).”<br><br>“Debilitating psychiatric illness may be a sensitive issue for patients and they may be uncomfortable with a crowd of students observing them; hence bedside tutorials on such conditions may be hard to come by. The virtual platform with filmed interviews with patients will allow all students to have equal learning opportunity.”<br><br>“Soft skills such as empathy and interviewing etiquette are better learnt by witnessing, and the video demonstration provided by the app/website is therefore useful.”

	“Overall, the Mastering psychiatry and its online platform have benefited the student population greatly, and made learning psychiatry, much easier than what I have initially thought it would be.”
Changed learning in the future	“The application and website, being portable in clinics and on the wards, allowed immediate access to information.”<br><br>“Collaboratively edited by clinical tutors, Mastering Psychiatry and its online platform has structured learning in a logical manner and includes comprehensive contents for exams and future clinical practice. Also the online nature of the application allows for constant updates with the latest developments in psychiatric practice, hence making one feel confident using it as a reference in the future without worries that its contents has become obsolete or irrelevant to current practice”<br><br>“The platform propels us to use accurate source of information, with validated information, instead of using search engines to search for explanation”
